# (Mis)recognition in the Therapeutic Alliance: The Experience of Mental Health Interpreters Working With Refugees in U.K. Clinical Settings

**DOI:** 10.1177/1049732320966586

**Published:** 2020-10-31

**Authors:** Hibah Hassan, Leda Blackwood

**Affiliations:** 1 University of Bath, Bath, United Kingdom

**Keywords:** interpreter, refugee, asylum seeker, therapeutic alliance, qualitative, reflexive thematic analysis, United Kingdom

## Abstract

Mental health interpreters play a crucial role in clinical support for refugees by providing a bridge between client and clinician. Yet research on interpreters’ experiences and perspectives is remarkably sparse. In this study, semi-structured interviews with mental health interpreters explored the experience of working in clinical settings with refugees. We conducted inductive analysis informed by a reflexive thematic analytic approach. Our analysis identifies interpreters’ pleasure in being part of people’s recovery, offset by the pain of misrecognition by clinicians that signals low self-worth and invisibility. Three sites of tension that create dilemmas for interpreters are identified: maintaining professional boundaries, managing privately shared information, and recognizing cultural norms. These findings are discussed in terms of the implications for clinicians working with interpreters, with a focus on the importance of a relationship of trust founded on recognition of the interpreters’ role and the unique challenges they face.

We are human beings. Human beings are complex, they are not machines that only do one action. And although it seems I am only doing one action, this action cannot happen without the other things. You know, other things have to support an action for it to be efficient. Otherwise, you don’t get it.

The United Nations Refugee Agency (2019) estimates that at the end of 2019, an estimated 79.5 million people worldwide were displaced due to conflict and human rights violations. Of these, 26 million are refugees and 4.2 million are people seeking asylum—18,519 of whom were granted some form of protection in the United Kingdom in 2019 (United Kingdom Home Office, 2018). Many of these people are at an increased risk of psychological distress as a result of traumatic experiences, including witnessing the murder of their family or friends, sexual or physical assault, torture, persecution, and separation from loved ones (Cleveland et al., 2014; Goodman, 2004; Porter & Haslam, 2005). Indeed, a recent systematic review of studies in Europe found that rates of post-traumatic stress disorder (PTSD) are higher in refugees and asylum seekers as compared with local populations (Priebe et al., 2016). As symptoms of PTSD negatively impact quality of life and are unlikely to remit without treatment (Lamkaddem et al., 2014; Priebe et al., 2009), there is a need for effective and accessible psychological support for refugees and asylum seekers.

Although mental health interpreters play a crucial role in clinical support for refugee populations (Priebe et al., 2016), some clinicians working in this area continue to believe that interpreters should provide only word-for-word translation in the therapeutic relationship between client and clinician (Engstrom et al., 2010; Hsieh et al., 2010; Mofrad & Webster, 2012). There is, however, increasing recognition of the practical limits to this and the need for a deeper understanding of the complex negotiations entailed in the interpreter’s role as *part of* the therapeutic process (Becher & Wieling, 2015; Dysart-Gale, 2005). Mental health interpreters bring their own knowledge, experiences, and values (Bhui & Morgan, 2007) that can sometimes facilitate the establishment of trust and rapport between client and clinician (Miller et al., 2005), and at other times create tensions (e.g., when there is asymmetry between their and the client or clinician’s understandings and goals; Mirdal et al., 2012). The refugee context, where there can be stigma associated with trauma, is one where these complexities may be magnified (Silove et al., 2017). Understanding the experiences of mental health interpreters is therefore crucial in enabling therapy in which the interpreter, as well as the client and clinician, are supported and acknowledged.

## Why Mental Health Interpreters Matter to Refugees

There is considerable research on the experiences of refugees accessing mental health services in the West, including in Europe (Maier & Straub, 2011; Markova & Sandal, 2016; Posselt et al., 2017), the United States (Miller et al., 2005; Morris et al., 2009; Shannon et al., 2015), and Australasia (De Anstiss & Ziaian, 2010; Valibhoy et al., 2017) that attests to the importance of interpreters in facilitating access. Alongside language barriers (Shrestha-Ranjit et al., 2020), much of this research attributes problems of access (and compliance with treatment) to cultural differences and unfamiliarity with the Western conception of mental health (e.g., Maier & Straub, 2011; Morris et al., 2009). Other research has been more equivocal about reliance on “cultural difference” explanations, drawing attention to professionals’ (and researchers’) cultural stereotypes (Kerbage et al., 2020; Kirmayer, 2006) and biases that privilege essentialized individual- and cultural-level explanations and solutions for distress over social, economic, and political ones (Melamed et al., 2019; Premji, 2019; Shannon et al., 2015; Tippens, 2017). Regardless of the explanatory framework, what is clear is that refugees themselves express fear about being misunderstood and offered unhelpful advice and support from professionals of different cultural or experiential backgrounds (Behnia, 2003; Bernardes et al., 2011; De Anstiss & Ziaian, 2010; Kerbage et al., 2020; Majumder et al., 2015), and have greater confidence in expressing themselves and being understood in the presence of an interpreter (Hadziabdic & Hjelm, 2014).

The role of interpreters from the refugee client perspective, however, is not always straightforward. For instance, there can be concerns about being judged when discussing sensitive issues (Hadziabdic & Hjelm, 2014), as well as concerns about confidentiality when working with interpreters from one’s own community (Palmer & Ward, 2007) and when working with compatriots who may have different political views and allegiances (Tribe & Lane, 2009). Notwithstanding the above complexities, there is now formal recognition of the need for interpreters in mental health services. For instance, in the United Kingdom, the National Institute of Clinical Excellence (2005) guidelines stipulate that foreign language interpreters should be provided to clients who have an insufficient command of the English language to access consultation and treatment.

## The Triadic Therapeutic Alliance

The client–clinician relationship is understood in terms of a therapeutic alliance, defined as a positive and collaborative relationship built on a shared commitment to the client’s goals (Miller et al., 2005). When an interpreter is introduced to a therapeutic system, this alliance shifts from dyadic to triadic, so creating a more complex relational dynamic (Bhui & Morgan, 2007; Schweitzer et al., 2013). The strength of the therapeutic alliance is particularly important in the refugee context where traumatized clients may be suspicious of authorities (Kerbage et al., 2020; Vasquez, 2007). Miller and colleagues (2005) found that when the interpreter and client shared a common background, trust and rapport were easily established early in the therapeutic relationship, and this was then transferred to the therapist (see also Mirdal et al., 2012; Williams, 2004).

The notion that interpreters can assist in establishing trust and rapport within the therapeutic relationship resonates with research on interpreters as relational mediators. For instance, a survey-based study by Boss-Prieto and colleagues (2010) investigated the perspectives of clinicians, clients, and interpreters on both the meaning and the strength of the therapeutic relationship. They found that clients and clinicians had very different views about what was important to developing the therapeutic relationship (e.g., assistance vs. guidance), but that interpreters shared some common perceptions with both clients and clinicians. They attributed this to interpreters being sensitive to and guided by their central role in mediating between the worlds of the client and the clinician.

Research examining clinicians’ perspectives on working with interpreters, however, is more equivocal about the benefits. Whereas some clinicians view interpreters as integral to relationship building through bridging cultural differences (Gartley & Due, 2017; Miller et al., 2005; Raval, 2005; Schweitzer et al., 2013; Yakushko, 2010), others do not and go so far as to describe interpreters as an “unfortunate necessity” and an “obstacle” to therapeutic contact with the client (Miller et al., 2005; p. 30). These more negative appraisals focus on two main areas of concern. First, some clinicians worry that reliance on interpreters may compromise the quality of communication; for instance, through inaccuracies in interpretation and the potential for bias and lost information (Engstrom et al., 2010; Mofrad & Webster, 2012). Second, some clinicians report feeling excluded in the triadic relationship and worry that the bond between the client and interpreter will compromise their own therapeutic alliance with the client (Hsieh & Hong, 2010; Raval, 2005). Research suggests that these relationship type concerns can in turn contribute to role ambiguities and anxieties about control of the treatment agenda (Mofrad & Webster, 2012), self-consciousness about having an interpreter witness their therapeutic work (Miller et al., 2005), and tensions between wanting more input from interpreters, while feeling uncomfortable about interpreters’ unsolicited interjections (Raval, 2003).

Although there is a substantial body of literature identifying challenges faced by clinicians and clients, and their perceptions about how the interpreter may help (or hinder) relationships between them (Engstrom et al., 2010; Raval, 2003), the experience of interpreters working in mental health services has received less attention (Hseih & Hong, 2010; Lor et al., 2019). One study examining the phenomenological worlds of nine refugee interpreters (Williams, 2004) identifies the considerable emotional work and the ambiguities and tensions that attend bridging the gap between the world of the client and that of the clinician. For the most part, however, research conducted with interpreters tends to focus on the interpreter’s *contribution* to the therapeutic relationship: This research highlights the support needed by refugee clients, including practical support, as well as interpreters’ own complex emotional responses to shared client–interpreter trauma history (Dubus, 2016; Gartley & Due, 2017; Johnson et al., 2009; Splevins et al., 2010).

The present study investigates mental health interpreters’ perspectives on the experiences of working with refugee clients that matter to them and how they make sense of these experiences. Ultimately, this study aims to enrich our understanding of what needs to be considered in providing support to mental health interpreters working in a triadic relationship with refugees and clinicians. Our premise is that sensitizing ourselves to the experiences and perspectives of this overlooked group of workers is necessary for critical and reflective clinical practices. But equally, we argue that by strengthening the triadic therapeutic alliance, this will also improve the quality of care received by refugee and asylum-seeking clients.

## Method

### Setting and Participants

The study was conducted at a United Kingdom NHS Psychology-based service for refugees and asylum seekers with a primary diagnosis of posttraumatic stress disorder (PTSD). The service follows a stepped pathway of care, including psychoeducation, behavioral therapies for depression, and trauma-focused cognitive behavioral therapy for PTSD. All stages of care may involve an interpreter who is typically culturally matched with clients. Most clients originate from the Middle East, predominantly from Iran, Iraq, and Syria; others come from East Africa, East Europe, and South Asia. Interpreters are booked through a third-party interpreting service and are paid an hourly rate.

The interviewer was a final year undergraduate psychology student on placement with the clinical team at the service. She recruited participants from the pool of interpreters who have experience working for the service. This was done in two ways. First, she outlined the study to interpreters during a biannual interpreter forum at which those interpreters most frequently used by the service are invited to discuss service improvement. Second, all clinical staff members were asked to give an information sheet to any interpreter they thought might be interested in sharing their experiences of work. In issuing the invitation, care was taken to ensure that interpreters understood that their participation was not expected, but if they did choose to participate, they would be paid the standard hourly rate.

Ten mental health interpreters between the ages of 30 and 55 years agreed to participate: seven females and three males (see Table 1). Interviewees all spoke at least one language other than English, including Albanian, Arabic, Bengali, Dari, Farsi, French, Kurdish Sorani, Latvian, Sylheti, Russian, and Tigrinya. Four interviewees came to the United Kingdom as refugees or asylum seekers themselves. Interviewees were formally trained and held a National Vocational Qualification Level 6, with a health element, or a Diploma in Public Services Interpreting (Health). All interviewees had worked for the service for at least 2 years prior to the research.

**Table 1.** Sociodemographic Characteristics of Participants.

Sociodemographic characteristics	*n*	%
Age range		
30–40	1	10
40–50	4	40
50+	5	50
Gender		
Female	7	70
Male	3	30
Number of languages spoken		
2	5	50
3	4	40
3+	1	10
Previously held refugee status		
Yes	4	40
No	6	60

In addition to working with the clinical team at the time of the study, the interviewer is a British-Pakistani, Muslim woman who observes hijab (Islamic code of modesty) and is conversational in Arabic. This placed her in a position of being both a potential outsider (attached to the clinical team), and insider (sharing language, culture, and experiences of minority British status with some interpreters). Accordingly, while there were some advantages in terms of understanding the context in which the interpreters were working and the wider context of being a member of an ethnic minority in Britain, there were also risks that needed to be considered. For instance, interviewees might assume shared knowledge and so not elaborate on their experiences, creating problems for data interpretation. More critically, they might have concerns about being judged and subjected to reprisals at work and so carefully manage their disclosures; or alternatively, they might see the interviewer as an ally and so over-disclose (see Dwyer & Buckle, 2009 on the insider-outsider researcher).

The study was approved by the University of Bath Psychology Ethics committee (approval number: 18-007). Participants were fully informed that the purpose of the research was to improve our understanding of interpreters’ experiences and perspectives, their data would be anonymized and stored securely, findings would be shared with the service in the form of a report written up for publication, and that they had the right to withdraw during and after the interview. All participants provided informed written consent prior to interview. The issues relating to the interviewer’s multiple positions and roles, and the potential risks for the interviewees and interviewer, were carefully considered in the development of the ethics application. We also, however, recognized the limits of ethical codes in addressing dynamic relational interactions and the importance, therefore, of the authors engaging in reflective research practice through ongoing discussions across the course of the interviews (de Smet et al., 2020).

### Design

Semi-structured, in-depth interviews were conducted to capture our interviewees’ subjective experiences and how they made sense of those experiences (Johnson, 2001). We decided against conducting focus groups because we wanted a range of perspectives and needed to be sensitive to potential power relationships in the employment context (Halcomb et al., 2007; Morgan, 1995). Interviewees were first asked, “How did you come to work as an interpreter with refugee clients”? The purpose was to ease into the interview and to understand something about the interviewee’s background and motivations for their work. We then moved to the main focus of the interview; interviewees were asked to tell stories about any experiences working as an interpreter (both positive and negative) that were memorable for making them think about their role in the therapeutic relationship (Smith et al., 2009). We made no assumptions about what experiences our participants might raise; rather, we sought to understand what they would consider significant and why. Accordingly, the interview was open-ended, with the interviewer simply probing for detail (e.g., What happened? Who was there? What did you do or want to do? Why do you think this happened?). The interviews lasted between 40 and 80 minutes.

### Data Analysis

The interviews were conducted in English and transcribed verbatim. We did not use specialist software programs for either transcription or analysis. Our analysis followed Braun and Clarke’s (2019) reflexive thematic analysis approach, which centers on researcher subjectivity and reflexivity and is consistent with a critical realist epistemology (Fletcher, 2017). According to this approach, codes and themes do not passively emerge from the data, but rather they are actively generated by the researchers. It is important that this is done not only systematically, but also in a manner that is fluid and recursive where the researchers move back and forth between stages of analysis. In the initial stage of the analysis, we immersed ourselves in the data to gain analytic insight and conducted initial inductive coding at a semantic level. Through a collaborative and reflexive process, the codes were further developed and collated based on patterns of shared meaning. The thematic analysis was also a collaborative process in which both authors shared and discussed their interpretations and returned to the transcripts and the coding in an iterative fashion. The authors worked toward consensus on each theme while paying particular attention to “negative cases” or data that might challenge the themes (Mays & Pope, 2000).

## Findings

Inevitably, interviewees’ experiences of working with refugees in a clinical context were complex; our interviewees had positive and negative stories to tell about interactions with both clients and clinicians. Moreover, reflecting on the nature of their freelance employment, our interviewees’ stories did not solely relate to the service where the interviews took place. Rather, their stories, and their sensemaking of these stories, drew on and contrasted with experiences across different mental health settings, including charities, hospitals, and GP practices. Across these stories, we identified two themes in the analysis. The first theme captures the tensions between loving the work and feeling that one’s contribution is not always recognized. The second theme captures dilemmas created by competing expectations in relationships with clients and clinicians: (a) maintaining professional boundaries, (b) managing privately shared information, and (c) recognizing cultural norms.

To preserve anonymity of our interviewees, we have removed all potentially identifying information from the extracts and use the pronoun “they” to refer to all interviewees.

### Theme 1: The Pleasure and the Pain of (Mis)recognition

In this first theme, we consider how interpreters’ sense of themselves and their self-worth is affected very differently by the relationships they have with clients and with clinicians. In the first subtheme, we see how the relationship with clients engenders a sense of recognition and achievement. In the second subtheme, there is not this same recognition from clinicians; but rather an experience of invisibility.

#### The experience of recognition and achievement

All interpreters in our sample spoke about enjoying their work and finding fulfillment through helping clients. There was a shared sentiment of finding the job highly rewarding and the suggestion that this might compensate for poor pay, as one interpreter observed, “certain jobs you don’t do it because of the money.” In talking about what made the job rewarding, some referred to extrinsic rewards such as recognition by clients, “At the end of the session, they ask for you again to come back. And that’s a big thing you’ve achieved,” and expressions of gratitude, “And sometimes they say duah (prayer) and when you hear, you know duah, it’s a very nice thing when you hear all this, you know.”

Beyond the expressions of recognition and gratitude from clients, many interpreters spoke of the intrinsic reward in seeing people get better: “When I see people who were broken, completely broken, and they come here, just a little, little bit of hope, I can’t even describe it. It’s when you see a patient come back to life again.” In the following extract, an interpreter speaks about a positive experience of their work:

I interpret for her for 6 years and she was terribly, terribly, terribly bad in trauma and suddenly she gets better and you can see her improvement getting better slowly, slowly, slowly. It was like, like I had an Oscar, to see her getting better. I love to do this job, to give what I can to help them.

In likening the experience of seeing a deeply traumatized client improve to winning an Oscar, this interpreter conveys a powerful sense of both joy and personal achievement; they clearly feel that they have contributed to this client’s recovery and take pride in this. Moreover, the fact that they have been involved with the same client for such a prolonged period also speaks to a sustained level of personal investment, being part of a gradual journey of recovery.

#### The experience of invisibility in the therapeutic relationship

The interpreters we spoke to were strongly identified with their work; they were proud and committed to the collective effort of helping clients with their recovery. Moreover, it was apparent that they had sometimes been working over many years with individual clients and, in some instances, perceived that they had more personal experience than clinicians. For instance, one interpreter comments on the awkwardness of needing to guide “beginner” clinicians “because you are someone who knows the cases better.”

Against this expressed commitment, what was striking in our data was the interpreters’ meta-perceptions of how they were regarded by clinicians (and other health professionals) and how these meta-perceptions spoke in some cases to a troubled power relationship. Interpreters spoke of the expectation that they should be invisible in the relationship between clinician and client: “We usually say that as a person, the interpreter is not there. Not there. . . The conversation is going on only between the clinician and the patient.” Whereas some believed that this was appropriate in the performance of their role, others expressed greater ambivalence and, in some cases, resentment:

They see themselves as a specialist, and I’m just talk, someone that just translates whatever is said to the other end. They don’t think that I also have my own brain. That I can process, and I have some experience about these things.

This interpreter’s perception of being reduced to their function of translating—to “just talk”—is experienced as an erasure of both their intelligence and their experience. The implication is the dismissal of what this interpreter might bring to the therapeutic relationship beyond mere translation. Others were explicit in explaining that they would not interpret culturally relevant information unless specifically asked to: “We are not allowed. If sometimes they ask us, otherwise we cannot interfere in cultural difference.”

A corollary of being invisible and reduced to “just talk” is also the nonrecognition of emotions. Indeed, this was regarded as a requirement of their role: “stay unattached. Not show any emotions and you know, you have to be a robot obviously.” However, while this interpreter describes the requirement to be a robot as “obvious,” what was clear in many of the interviews was how difficult this was, and how the fiction of interpreters remaining unattached may render their own suffering invisible. Here an interpreter describes struggling to remain emotionally neutral when hearing about a client’s trauma following the loss of her parents:

I was missing my parents a lot and I couldn’t help myself. I left the room for a couple of minutes and then I came back and apologised and yeah, because her mum died in the sea, and she said, “if you don’t see them, you can’t believe that they are dead.” And because I haven’t seen my family for a very long time, it was very touching.

This interpreter highlights a fundamental challenge of working with trauma, the effects of which may be amplified when interpreters are reminded of their own experience of trauma (see also Johnson et al., 2009). In this interpreter’s expression of remorse, we can see the professional expectation, presumably shared with the clinician, to manage one’s emotions. What is perhaps most poignant here is that we are left with the figure of the interpreter whose own trauma goes unacknowledged.

The perceived expectation to remain emotionally neutral in the face of hearing traumatic stories was echoed by other interpreters, one of whom described it as “dehumanising” and went on to explain,

I think no one really cares how the interpreter feels . . . I think it would be good if the therapist would ask “Are you okay,” “Do you need a five-minute break or a sip of water or something?”

### Theme 2: Caught Betwixt and Between Client and Clinician

The establishment of trust is critical to facilitating trauma-focused therapy (Chouliara et al., 2017) and, in triadic interactions, must be developed between all three parties. In this study, interpreters described clients first placing trust in them before trusting the clinicians, leading to close client–interpreter relationships, as explained by one interpreter: “I’m just here for he or she to trust me first, and then the doctor or the physician” (see also Mirdal et al., 2012). This theme explores three main dilemmas that interpreters face as a consequence of their complex role in the triadic relationship: a role that creates tensions between the expectations of clinician and client.

#### Difficulty maintaining professional boundaries

In the previous theme, we saw how deeply affected interpreters were by their clients’ experiences and the strength of the relationship that might arise from shared understanding and long-term involvement. But the strength of this relationship may also pose problems. The consequence of this was sometimes becoming the repository of greater trust (“they trust me, because they know I know where the’re coming from”), which in turn occasioned a sense of responsibility and accountability to the client (“Yeah, they trust the doctor, but they trust me more that I am speaking on their behalf”). Here, an interpreter describes the tensions entailed in these strong relationships:

Interpreter: It’s a very sensitive job, and they are very attached to us, they start to be attached to us, which is in a way good and a way not good.

Researcher: How so?

Interpreter: In a way it is good, they will trust us, they will find us more friendly, more helping, you know. In other way, when they are very attached to us they start to ask us more questions, more outside the session and sometimes you can’t answer. And sometimes they ask for phone number to call us, I said “no we are not allowed,” so there is a limit, but it is difficult for us.

Although clearly recognizing the therapeutic value of establishing a relationship of trust with clients, this interpreter also recognizes the potential blurring of professional boundaries when clients misinterpret the relationship as friendship. Boundaries also become blurred when clients expect interpreters to advocate for them:

They expect me to help them and advise them. But I’m just an interpreter . . . They feel because I’m speaking their language, we are on one team. But I am impartial. Completely impartial.

Again, we see the potential perils associated with shared understanding; the expectation that one is “on the same team” and the need for the interpreter to assert their professional impartiality. Moreover, in protesting that “I’m just an interpreter,” there is the suggestion that perhaps one is considered to hold greater authority than is the case. Others too spoke of tensions created by clients wanting help with negotiating the system: “Yes, they think I will help them with how to do benefits, or how to speak or to forward, or to jump the queue and I said no, this is the system here.”

#### Managing information shared privately

A key benefit of a close client–interpreter relationship is clients’ greater willingness to disclose (Miller et al., 2005). But, from the perspective of the interpreters in our sample, this was attended by some peril where clients revealed information in the absence of clinicians and with the express expectation that the information remain “just between you and I.” One interpreter spoke of clients’ trust in them leading to revelations of untruths told to clinicians: “I don’t know, even sometimes when they make up something, they tell me afterwards. ‘Oh, I said that because of that.’ They trust me. They feel closer to me.” On one hand, this experience may be viewed as testimony to the strength of the client–interpreter relationship. On the other hand lies the expectation that the interpreter will not betray the client’s confidence by reporting to the clinician what has been said.

Here, an interpreter describes a situation in which they are interpreting for a married couple where the wife has mentioned the husband’s anger in front of the clinician:

And then when the clinician left the room to print another questionnaire, he said to his wife “you shouldn’t have said that to her.” Because they think here in this country, once you mention that you have any problems with the children, they will take them from you. And he told the wife, “now when you get the questionnaire you have to say everything positive and don’t mention anything else.”

In this situation, the information revealed is presumed to be kept between the interpreter and the clients—not to be shared with the clinician due to fear of the local welfare system, the mistrust of which is extended to the clinician, but not toward the interpreter. Similar situations were reported by other interpreters who recounted experiences of clients confiding in them in waiting rooms or after clinical sessions. For example, one described clients confiding in them about domestic abuse along with their concerns that if shared, “the therapist comes and just confronts the man, the husband, then they will be in a very difficult situation.”

In such situations, where information had been disclosed privately, persuading the client to share the information with the clinician was sometimes the first strategy: “I say you’d better let them (the clinician) know and ask them not to confront your husband and see whether they have any solutions.” Other interpreters spoke of basing their decision to disclose on their perceived judgment of risk of harm to the client or others. This judgment of risk was clearly not straightforward; for instance, interpreters spoke of needing to consider what may have been shared as part of building rapport and whether or not it was relevant to the therapeutic process.

Moreover, as the following example shows, the perceived betrayal of the client’s trust could have negative consequences not just for the therapeutic relationship but for interpreters too. Here, a situation is described in which a male client was given an injunction barring him from entering his wife’s property:

I just saw the husband coming from the house, and he just came towards me and said, “don’t say that I’m here!” But I should say. I should say. I told to the social worker. After that, apparently, they have to document it and each time I saw him, he was shouting, and I realised he wants to strangle me because the aggression on his face. It was awful.

Here we see an interpreter who has withstood what might be seen as considerable pressure to remain silent (i.e., being confronted by a male who has an injunction for violence) and shared information with a social worker. They are subsequently subjected to recurring instances of anger and visible aggression in the performance of their work.

#### Tensions between abiding by and violating cultural norms

All interpreters spoke of the challenges created by clinicians’ questions that required they violate shared (client–interpreter) norms, particularly in relation to sensitive topics that trigger strong feelings of shame in their communities. What was felt to be challenging was that the questions could appear to be coming from the interpreter, and thus risk jeopardizing the client–interpreter relationship. Such questioning could be regarded as at best revealing cultural incompetence (“I am supposed to know. I’m supposed to know where that person is coming from, because we are from the same culture”) and, at worst, violating moral codes of conduct (“It is difficult and its rude, they take it as rude sometimes”).

But it is not just in the context of facilitating communication between clinician and client in the clinical setting that interpreters encounter difficulties. This is exemplified by an account of having to turn down an invitation to enter a client’s home:

For example, I had to do home visit. I was early, and I stood outside the gate. The client saw me, he came out and said, “come in,” I said, “I can’t.” You know, you can’t go inside without the clinician. He got angry, he said, “you angry at me,” he said, “you think you’re better than me.” I left. In our culture, you go into someone’s house when they ask you. (Pause). I wanted to be polite.

Two things are noteworthy in this example. First, the interpreter’s ambivalence is reflected in their regret at being unable to participate in a practice of politeness, which they understand in terms of shared cultural identity. This challenges the view that the client–interpreter relationship is completely one-sided and highlights the impact culturally normative expectations may have on the interpreter as well as the client. Second, the misunderstanding of the interpreter’s reason for not entering his home potentially compromises the client–interpreter relationship. At least from the interpreter’s perspective, the client’s angry response is because they understand the interpreter’s failure to abide by shared cultural norms of politeness as a deliberate choice. This incident resonated with other interpreters’ accounts of being blamed for their behavior due to the expectation that they should “know better.” Importantly, inherent in being blamed for one’s behavior is the assumption of agency and a level of authority, which interpreters themselves may not feel.

One strategy for averting damage to the client–interpreter relationship is to make explicit one’s awareness that a shared cultural norm is potentially being violated and that responsibility for this lies with the clinician. This interpreter describes their typical approach (“not here,” but in other clinical contexts) to asking whether a Muslim client drinks alcohol:

I mean, when they ask that sort of question, not here, but I usually say, I explain to clients that they ask this question to everyone. “I know that you are, I know your answer, but I have to translate and ask . . .” I say, “I’m sorry I have to ask this question, they ask this question to everyone.”

The strategy employed by this interpreter involves signaling cultural understanding and trustworthiness to the client by expressly acknowledging Islamic beliefs around consuming alcohol. Their apology indicates awareness of and potential regret at endangering this trust. Moreover, by reiterating that it is “they” who ask such questions, the interpreter aligns with the client and, in doing so, keeps a distance from the faux pas committing clinician. Crucially, this strategy for navigating cultural impasses presents two potential problems. First, it entails affirmation of an alliance between client and interpreter that is defined by opposition to the clinician’s questionable cultural competence and morality. Second, the suggestion that “I know what your answer is” to a question that is culturally sensitive may make it difficult for clients to admit violation of assumed cultural norms.

## Discussion

We opened this article with an eloquent call from one of our participants to be humanized and to have the challenges and complexities she faces in her job acknowledged. This call captures something that threads through our analysis: a group of workers who identify with and are dedicated to what they do, but who experience a denial of this identity and dedication, and consequently, feel atomized and unsupported in the performance of their work. Misrecognition and denial of important identities that provide a basis for one’s sense of worth are painful experiences and have been shown to negatively affect the individual’s self-esteem and agency (Blackwood et al., 2015; Fidan, 2017) and to compromise group goals (e.g., through undermining trust and collective effort; Blackwood et al., 2015; Renger & Simon, 2011). Reflecting these processes, what was striking in our interviews were the apparent difficulties and absence of trust in sharing information with clinicians as well as the perceived lack of support from clinicians.

The interpreters in our study cataloged numerous instances where they needed to make decisions that were consequential for the client (and sometimes themselves), such as around safeguarding and risk—including determining what may or may not constitute clinical risk of harm to self or others. But it was not always evident that there was a trusting professional relationship with clinicians such that they would involve them in their decision-making. Indeed, some interpreters described work contexts where they were actively discouraged from engaging in the wider clinical conversation. Consequentially, not only may the experiential knowledge of the interpreter be overlooked, but also their ability to act as bridge between client and clinician, by sharing cultural expertise, may be hindered (Miklavcic & LeBlanc, 2014). When the interpreter feels constrained in sharing knowledge and experience, this may present a substantial barrier to the therapeutic alliance and, in some cases, may even inadvertently compromise the validity of clinical information relayed to the clinician.

What may be particularly germane to the issue of trust is the absence of support and recognition in relation to the emotional challenges entailed in dealing with traumatic content (Butler, 2008; Holmgren et al., 2003). Our findings highlight three reasons why this may be particularly egregious and consequential for the interpreter–clinician relationship. First, complex emotions are regarded as the hallmark of what makes us human and to be denied emotions is considered a form of dehumanization (Haslam, 2006). Second, the denial of one’s emotions is being done by those who are trained to recognize and respond to emotion, thus emphasizing one’s dehumanized status and the sense that “no one really cares how the interpreter feels.” Third, interpreters may share the normative expectations of their professional role and so experience shame in their emotional responses and participate in their own self-silencing (Van Kleef et al., 2015).

### Limitations and Future Directions

Our data cannot speak to the representativeness of the experiences described here and nor to whether and how interpreters’ experiences might differ across contexts. This was a small study conducted with interpreters who were recruited at a single mental health service. Almost all interviewees were those recommended by clinicians at the service or those who attended the biannual forum; hence, it is important to consider that these were interpreters who were perhaps more experienced and favored by the service. Moreover, our interviewees drew on experiences across a variety of organizational settings and the particular context they were referring to was not always clear. Further research is needed that takes account of the diversity among interpreters, as well as the diversity of settings in which interpreters work.

Our data also cannot speak to the veracity of our interviewees’ claims about how they are regarded and (de)valued by clients and clinicians. If we had interviewed clinicians, we almost certainly would have heard very different perspectives. This said, there is a substantial body of literature that speaks to the importance of meta-perceptions (beliefs about what others think), particularly for those in a low status relationship (Lammers et al., 2008), and the consequences of meta-perceptions for shaping identity and one’s sense of how one can and cannot act (Wakefield et al., 2013). This work reminds us of the importance of considering how asymmetries of power affect perspective-taking within professional relationships. To deepen our understanding of these processes and the interactional dynamics involved, what is needed as a first step is ethnographic research. Ideally, this would involve the researcher observing both dyadic and triadic interactions between clients, interpreters, and clinicians over time and examining the policies and practices that structure these relationships. For instance, something that was hinted at in our data, but which we were unable to explore, is the potential impact of the nature of the employment relationship and what this means for how interpreters are included and supported across the range of organizational settings in which they work.

### Conclusion and Recommendations

Our observations are consistent with those of Becher and Wieling (2015) who suggest that “silencing” interpreters in the therapeutic alliance oftentimes reflects clinicians’ concerns around maintaining power in the clinical setting. However, to the extent that the interpreter’s role beyond mere translation is valued (i.e., building trust and shared understanding between client and clinician), this silencing of interpreters and rendering them invisible presents a substantial barrier to the therapeutic alliance (Gartley & Due, 2017; Miklavcic & LeBlanc, 2014; Williams, 2004). Moreover, even where this therapeutic alliance is not valued, clinicians’ failure to understand the complexity of the context in which interpreters work (i.e., existing beyond what is merely “said” in therapy) is potentially damaging to both interpreter and client (see also Williams, 2004, on this point).

We wish to conclude with a number of recommendations for practitioners. First, the findings of this study underscore the importance of clinicians being aware of the complexity of the interpreters’ role and the challenges they face, the limitations of their own understanding of interactional nuances that may arise within therapy, and the dynamics entailed in triadic relationships (Bhui & Morgan, 2007; Miller et al., 2005). Accordingly, training in working with interpreters as well as broader implications of cultural and experiential diversity for clinical practice needs to be a core part of professional training and development for all psychologists. Second, we echo arguments made elsewhere (e.g., Becher & Wieling, 2015; Butler, 2008; Fidan, 2017; Holmgren et al., 2003; Miller et al., 2005; Raval, 2005; Tribe & Lane, 2009; Williams, 2004) that structured support and supervision is needed to develop and recognize interpreters’ professional identities and commitment, to collaborate in the development of strategies within the therapeutic alliance, and to ensure that interpreters’ own mental health needs are recognized and supported.

In sum, this research highlights the need to bring mental health interpreters in from the cold. Central to this is developing strong clinician–interpreter relationships based on recognition of shared goals (Hsieh et al., 2010), respect for the complexity of interpreters’ roles and challenges (Becher & Wieling, 2015; Miller et al., 2005), and embedding these principles in both training and in organizational structures and practices (Tribe & Lane, 2009).
